# Positive Feedback Cycle of TNFα Promotes Staphylococcal Enterotoxin B-Induced THP-1 Cell Apoptosis

**DOI:** 10.3389/fcimb.2016.00109

**Published:** 2016-09-21

**Authors:** Xiaopeng Zhang, Weilong Shang, Jizhen Yuan, Zhen Hu, Huagang Peng, Junmin Zhu, Qiwen Hu, Yi Yang, Hui Liu, Bei Jiang, Yinan Wang, Shu Li, Xiaomei Hu, Xiancai Rao

**Affiliations:** Department of Microbiology, College of Basic Medical Sciences, Third Military Medical UniversityChongqing, China

**Keywords:** staphylococcal enterotoxin B, atopic dermatitis, apoptosis, THP-1 cells, tumor necrosis factor alpha

## Abstract

Staphylococcal enterotoxin B (SEB) has been demonstrated to be of importance in *Staphylococcus aureus* related diseases, such as atopic dermatitis (AD). Dysregulated apoptosis in AD is remarkable, and SEB can induce apoptosis of various cell types. However, the mechanisms by which SEB induces apoptosis and influences disease processes remain unclear. In this study, the recombinant SEB-induced THP-1 monocyte apoptosis was demonstrated in the absence of preliminary cell activation in a time- and dose-dependent manner. SEB could up-regulate the expression of tumor necrosis factor alpha (TNFα) in THP-1 cells and induce apoptosis via an extrinsic pathway. TNFα could in turn increase the expression of HLA-DRa, the SEB receptor on the cell surface. As a result, a positive feedback cycle of TNFα was established. TNFα expression and SEB-induced apoptosis were decreased by knocking down the expression of either HLA-DRa or TNFR1. Therefore, the feedback cycle of TNFα is crucial for SEB functions. This work provides insights into the mechanisms of SEB-induced monocyte apoptosis and emphasizes the major role of TNFα in future related studies.

## Introduction

Bacterial superantigens are a family of potent immunostimulatory exotoxins that activate T lymphocytes. As a major superantigen-producing pathogen, *Staphylococcus aureus* can secrete more than 20 distinct superantigens correlated with various human diseases and disorders, such as food poisoning, toxic shock syndrome, Kawasaki disease, and atopic dermatitis (AD; Xu and Mccormick, 2012). AD is a chronic, recurrent, and pruritic skin disease, which is closely related to *S. aureus* colonization and infection. At least 80% of *S. aureus* strains isolated from patients with AD produce superantigens (Ong and Leung, 2010). Patients with severe corticosteroid-insensitive AD harbor *S. aureus* strains that produce a significantly high number of superantigens (Schlievert et al., 2008). These findings imply the important roles of staphylococcal superantigens in AD.

Staphylococcal superantigens include staphylococcal enterotoxins (SEs), staphylococcal enterotoxin-like proteins, and toxic shock syndrome toxin-1 (Pinchuk et al., 2010). As the most well-characterized superantigen, staphylococcal enterotoxin B (SEB) is expressed by many of the *S. aureus* isolates and correlated with increased AD severity (Raap et al., 2008). Staphylococcal superantigens bind to natural receptors, namely, T cell receptor (TCR) and type II major histocompatibility complex (MHCII) molecules. SEB can ligate with the β chain of TCR to induce hyper-inflammatory responses and auto-immune reactions, and with the HLA-DRa of MHCII to induce cell apoptosis (Xu and Mccormick, 2012). Dysregulated apoptosis plays an important role in the pathological process of AD (Xu and Mccormick, 2012). T cells from patients with AD are more sensitive to SEB-induced apoptosis compared with that of the healthy individuals, which is correlated with the severity of this disease (Kędzierska et al., 2005). Peripheral blood mononuclear cells from patients with AD are also sensitive to SEB-induced apoptosis (Sohn et al., 2003). SEB causes T lymphocytes to undergo activation-induced cell death, which involves TCR binding and FAS expression (Ulett and Adderson, 2006). However, whether SEB can induce the apoptosis of monocytes and influence the pathology of *S. aureus*-induced diseases remain unclear.

MHCII molecules have been recognized as antigen-presenting structures expressed on antigen presenting cells (APCs). Since the late 1990s, MHCII molecules have been demonstrated to transduce extracellular signals from cell proliferation and maturation to apoptosis (Al-Daccak et al., 2004). Mouse anti-HLA-DR monoclonal antibody (mAb) L243 can induce human monocytes death via a caspase-independent protein kinase C (PKC) activation pathway (Thibeault et al., 1999; Castaigne et al., 2002). However, mAb may function differently from the natural ligands of MHCII molecules, such as staphylococcal enterotoxin A (SEA) and SEB, *in vivo*. Indeed, although SEA and mAb L243 share a common epitope on HLA-DR, their abilities to induce apoptosis of monocytes through MHCII molecules are different (Gross et al., 2006).

In this study, the SEB-induced apoptosis of THP-1 human monocytic cell line was examined. Our results indicated that SEB can induce THP-1 cell apoptosis via an extrinsic pathway, and a positive feedback cycle of TNFα likely triggers and promotes apoptosis.

## Materials and methods

### Expression and purification of SEB

The coding sequence of SEB was amplified through PCR from the genomic DNA of *S. aureus* strain XQ (GenBank accession number: NZ_CP013137; Rao et al., 2015) with primer pairs BamHI-F: 5′-CGCGGATCCATGTATAAGAGATTATTTA-3′ and XhoI-R:5′-CCGCTCGAGCTTTTTCTTTGTCGTAA-3′. The PCR products were isolated through 1% agarose (m/v) gel electrophoresis, purified with a DNA purification kit (TaKaRa, Liaoning, China) in accordance with the manufacturer's instructions, digested with *Bam*HI and *Xho*I, and ligated into pET30a expression plasmid (Merck Millipore, Hong Kong, China). The correct recombinant plasmid carrying the *seb* gene was confirmed through restriction enzyme analysis and DNA sequencing and was designated as pET-SEB. The 6 × His-tagged SEB was expressed in 1 L of *Escherichia coli* strain C43 (2nd lab, Shanghai, China) carrying pET-SEB induced with 5 μM isopropyl-d-thiogalactopyranoside (IPTG) at 25°C for 8 h. The cells were then centrifuged at 10,000 × g for 15 min, washed once with phosphate-buffered saline (PBS, pH 7.0), re-suspended in PBS with 0.5 mM PMSF for ultrasonic disruption, and centrifuged at 16,000 × g, 4°C for 30 min. The supernatant protein was purified by His-Mag Sepharose immobilized metal affinity chromatography (GE Healthcare Life Sciences, Pittsburgh, PA, USA) with balance buffer PBS (pH 8.2) and elution buffer PBS containing 500 mM imidazole (pH 8.2). The recombinant SEB was further purified by using an endotoxin-removing gel (Pierce, Rockford, IL, USA) to avoid potential contamination of LPS. The buffer was concentrated and changed to PBS (pH 7.0) by Amicon Centrifugal Filter Units (Merck Millipore). Protein concentration was determined with a Bradford assay (Beyotime Biotechnology, Jiangsu, China) and Bull Serum Albumin (BSA) was used as the standard. The total yield of SEB was approximately 4.5 mg/L for one preparation. The LPS concentration of the protein stock was lower than 2 EU/ml, as determined by a tachypleus amebocyte lysate test (Horseshoe Crab Reagent Manufactory Co., Ltd., Xiamen, China). Thus, the final concentration of LPS was lower than 0.08 EU/ml in the experimental assays.

### Cell culture

The human monocytic cell line THP-1, a generous gift from Professor Shen of Chongqing Medical University, was authenticated by short tandem repeat (STR) identification in Microread Genetics Co., Ltd. (Beijing, China). This cell line showed 93.3% similarity to the ATCC human cell line THP-1 and did not exhibit cross-contamination. Unless otherwise indicated, the cells were cultured in RPMI 1640 medium (Thermo Fisher Scientific, Rockford, IL, USA), supplemented with 10% (*v*/*v*) fetal bovine serum (Thermo Fisher Scientific), 2 mM glutamine, and 0.1% (*v*/*v*) of β-mercaptoethanol (Thermo Fisher Scientific) in a T25 flask at 37°C in 5% CO_2_, and the culture was transferred to 24-well-plates (2 × 10^5^ cells, 0.6 ml medium per well) for the subsequent assays.

For cell activation, 1 μg/ml of IFN-γ (6 μl/well, R&D Systems, Minneapolis, MN, USA) or LPS (6 μl/well, Promega, Madison, Wisconsin, USA) was added 24 h before the treatment, as described previously (Thibeault et al., 1999; Castaigne et al., 2002), and 6 μl/well PBS was added to the control wells. For cell differentiation, phorbol-12-myristate-13-acetate (PMA; Promega) was utilized in accordance with previously described methods with modifications (Genin et al., 2015). In brief, the PMA stored in DMSO was diluted to 6 μM (1:100) with fresh RPMI 1640 medium before use. The cells cultured in 24-well-plates were treated with 30 nM PMA (3 μl/well), and the cells in the wells with 3 μl of RPMI 1640 medium containing 1% (*v*/*v*) DMSO served as controls. The final concentration of DMSO was 0.005% (*v*/*v*). After 48 h of differentiation, the medium was changed before the subsequent assays were conducted. For caspase inhibition, a caspase inhibitor Z-VAD-FMK (Beyotime Biotechnology) stored in DMSO was diluted to 2 mM (1:10) by RPMI 1640 medium and added to cells (6 μl/well), and the cultures in the wells with 6 μl of RPMI 1640 medium containing 10% (*v*/*v*) DMSO served as controls. The final concentrations of Z-VAD-FMK and DMSO were 20 μM and 0.1% (*v*/*v*), respectively. In accordance with the manufacturer's recommendation, Z-VAD-FMK was added 30 min before subsequent assays were performed.

### Apoptosis analysis

Cells were stained with Annexin V-FITC apoptosis analysis kit (Sungene Biotech, Tianjin, China) and analyzed through flow cytometry (Partec, Görlitz, Germany) in accordance with the manufacturer's instructions. In a dose-dependent apoptosis assay, THP-1 cells (540 μl medium per well) were treated with 60 μl/well PBS containing different concentrations of SEB (0, 50, 100, 200, and 500 μg/ml) for 36 h. In a time-dependent apoptosis assay, the cells (576 μl medium per well) were treated with 20 μg/ml SEB (24 μl/well) or 24 μl/well PBS for 2, 4, 8, 12, 24, 36, 48, and 72 h. Otherwise, the cells were treated with 20 μg/ml SEB (24 μl/well) or 24 μl/well PBS for 36 h. For anti-TNFα neutralization assay, 5 μg/ml (3 μl/well) anti-TNFα (Abcam, Inc., Shanghai, China) or an isotype control IgG (Abmart, Inc., Shanghai, China) was added 30 min before SEB treatment was administered.

The cells in each well were collected, washed once with PBS, re-suspended in 100 μl of staining solution containing 5% (*v*/*v*) Annexin V-FITC and 5% (*v*/*v*) PI, and incubated in the dark for 15 min at room temperature. The cells were then analyzed through flow cytometry and with Flowjo7.6 (Treestar Inc., San Carlos, CA, USA). For each determination, at least 20,000 cells were analyzed. The cells stained with Annexin V+/PI− were considered as early apoptotic cells, and Annexin V+/PI+ were regarded as late apoptotic and necrotic cells (Pietkiewicz et al., 2015). Total cell death corresponded to all cells positively stained with PI.

### Caspase activity assay

Caspase activity was determined by using caspase activity assay kits (Beyotime) in accordance with the manufacturer's instructions. Briefly, 2 × 10^6^ cells were inoculated in 6-well-plates (1920 μl medium per well) and treated with 20 μg/ml SEB (80 μl/well) or 80 μl/well PBS for 36 h. Afterward, the cells were washed once with PBS, re-suspended in 100 μl of lysis buffer in an ice bath for 30 min, and centrifuged at 16,000 × g, 4°C for 15 min. The total protein in the supernatant was subsequently determined via Bradford's method (Beyotime), and 90 μl was transferred to 96-well-plates (30 μl/well) and incubated at 37°C for 2 h with 60 μl/well testing buffer and different substrates (Ac-DEVD-pNA for caspase-3, Ac-IETD-pNA for caspase-8, and Ac-LEHD-pNA for caspase-9; 10 μl/well). The enzyme-catalyzed release of chromophore pNA was quantified by using a spectrophotometer (SoftMax Pro, Molecular Devices, Sunnyvale, CA, USA) at 405 nm. Caspase activity was calculated as international units per μg of sample protein concentration (IU/μg).

### Quantitative real-time PCR

Approximately 2 × 10^6^ cells were inoculated in 6-well-plates (1920 μl medium per well) and treated with 20 μg/ml SEB (80 μl/well) or 80 μl/well PBS for 36 h. Afterward, the cells were collected and washed once with PBS. Total RNA was extracted by using a TriPure isolation reagent (Roche Applied Science, Indianapolis, IN, USA). RNA quality was confirmed through agarose electrophoresis and absorbance ratio (A260/280) determination (1.96–1.98, Nanodrop ND-1000, Thermo Fisher Scientific). Total RNA (500 ng) was treated with DNase (TaKaRa) at 37°C for 30 min followed by addition of 10% (*v*/*v*) stop solution. The resulting mixture was then incubated at 65°C for 10 min to eliminate possible DNA contamination. Then, cDNA was synthesized using a PrimeScript RT reagent kit (TaKaRa) from 500 ng of RNA with random primers in a 10 μl reaction mixture. The qRT-PCR was performed using SYBR Premix Ex *Taq* II (TaKaRa) on a CFX connection qPCR System (BioRad, Hercules, CA, USA). Each reaction volume was 20 μl, with 10 μl SYBR Premix Ex Taq II, 1 μl sense primer (10 μM), 1 μl antisense primer (10 μM), 8 μl diluted cDNA template (1:80 by RNase-free water). Sense and antisense primers were designed with Primer Premier Software (Palo Alto, CA, USA) or as described elsewhere (Wang et al., 2013; Table S1). The efficiency of each primer pair was determined on the basis of standard curves, and relative expression levels were normalized to GAPDH. The qRT-PCR procedure was: (i) denaturation: 95°C for 3 min; (ii) thermocycling for 40 times: (95°C for 10 s, 55°C for 10 s, 72°C 30 s); (iii) the melting curve determination. At least three technical repeats and four biological repeats were performed for analysis.

### Western blot

THP-1 cells were treated with SEB and lysed, and their total protein content was determined as described in Caspase Activity Assay Section. Approximately 25 μg of protein was separated through sodium dodecyl sulfate-polyacrylamide gel electrophoresis (SDS-PAGE) and transferred to a polyvinylidine difluoride (PVDF) membrane (Mini Trans-Blot Cell, BioRad). The PVDF membrane was blocked with 5% (*m*/*v*) non-fat milk (Boster, Wuhan, China) in TBST buffer (50 mM Tris, 138 mM NaCl, 2.7 mM KCl, pH 8.0, with 0.05% (*v*/*v*) Tween 20; ZSGB-BIO, Beijing, China) for 1 h and incubated in anti-HLA-DRa (1:200; Santa Cruz Biotechnology, Paso Robles, CA, USA), anti-TNFR1 (1:200; Santa Cruz), and anti-β-actin (1:1000, Boster) diluted in 5% (*m*/*v*) non-fat milk at 4°C overnight. The membrane was washed five times with TBST, incubated in goat-anti-mouse secondary antibody conjugated with horseradish peroxidase (HRP; 1:3000, Boster) diluted in 5% (*m*/*v*) non-fat milk for 1 h, and washed five times with TBST. Immunoreactivity was visualized by using a SuperSignal West Dura substrate (Thermo Fisher Scientific). Bands were detected by using ChemiDoc Touch Imaging System (BioRad) and densitometrically analyzed with ImageJ 1.51c (Wayne Rasband, National Institutes of Health, USA).

### Inhibition of HLA-DRa and TNFR1

The siRNA knock-down was conducted in accordance with the manufacturer's instructions and previously described methods with modifications (Zhao et al., 2012). In brief, 32 μl of siRNAs (HLA-DRa siRNA, TNFR1 siRNA, and non-target control siRNA; 10 μM; Santa Cruz) were diluted in 400 μl of transfection medium (Santa Cruz), mixed with 400 μl of transfection medium containing 24 μl of transfection reagent (Santa Cruz; 856 μl in total for each siRNA reagent), and incubated in the dark at room temperature for 30 min. The cells (2 × 10^6^) were washed once with 2 ml of transfection medium, re-suspended in the siRNA reagent, and incubated at 37°C for 6 h in a CO_2_ incubator. The siRNA reagent was removed and replaced with 2 ml of fresh culture medium for another 24 h.

The efficiency of siRNA inhibition was confirmed through Western blot. To increase the basal expression level of HLA-DRa, we added 1 μg/ml IFN-γ to both HLA-DRa siRNA-inhibited cells and control cells.

### Determination of TNFα with ELISA

The secreted TNFα was quantified by using a human TNFα ELISA kit (Elabscience, Hubei, China) in accordance with the manufacturer's instructions. Briefly, the cells inoculated in 24-well-plates were treated with 20 μg/ml SEB (24 μl/well) or 24 μl/well PBS for 36 h and centrifuged at 200 × g for 5 min. The supernatants were transferred to 96-well-plates (100 μl per well) and incubated at 37°C for 90 min. Afterward, 100 μl of biotinylated detection antibody was added and incubated at 37°C for 1 h. The wells were aspirated and washed thrice, and 100 μl of HRP conjugate was added and incubated at 37°C for 30 min. The wells were subsequently washed five times, and 90 μl substrate solution was added and incubated at 37°C for 15 min. Then, 50 μl of the stop solution was added. The OD of each well was determined by using a spectrophotometer (Molecular Devices) at 405 nm. The concentration of TNFα was calculated as picogram per milliliter (pg/ml) on the basis of the standard curve.

### Statistical analysis

Data were analyzed using SPSS 18.0 (Chicago, IL, USA). Student's *t*-test and one-way ANOVA were performed to compare categorical variables. All analyses were two-tailed, and a *P*-value of <0.05 was considered statistically significant.

## Results

### SEB induced THP-1 cell apoptosis even without cell activation

**Figure 1:**
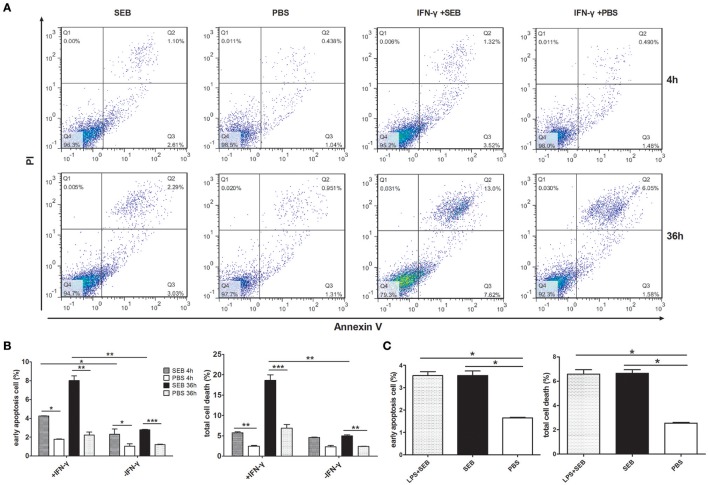
**SEB induced THP-1 cell apoptosis even without cell activation**. THP-1 cells (2 × 10^5^ per well in 24-well-plates) with or without activation of 1 μg/ml IFN-γ for 24 h were treated with 20 μg/ml (24 μl/well) SEB or 24 μl/well PBS for 4 or 36 h as indicated. **(A)** Apoptosis were measured by Annexin V/PI staining and flow cytometry. Cells stained with Annexin V+/PI− were considered as early apoptotic cells, and Annexin V+/PI+ were regarded as late apoptosis and necrotic cells. Total cell death corresponded to all of the cells positively stained with PI. **(B)** Quantitatively analyzed data are presented as means ± *S.D*. (*n* = 4), ^*^indicated *P* < 0.05, ^**^indicated *P* < 0.01, and ^***^indicated *P* < 0.001. **(C)** Quantitative analysis for THP-1 cells treated with 20 μg/ml (24 μl/well) SEB for 36 h, with or without a previous activation by 1 μg/ml LPS. Data are presented as means ± *S.D*. (*n* = 3), ^*^indicated *P* < 0.05 vs. PBS control.

IFN-γ is commonly used to activate THP-1 cells prior to experiments because MHCII expression varies among cells and activation states (Thibeault et al., 1999; Castaigne et al., 2002; Gross et al., 2006). We also performed this procedure and found that the percentage of apoptotic cells was significantly increased, especially at 36 h post-treatment, when THP-1 cells were activated by IFN-γ for 24 h before they were treated with recombinant SEB. However, SEB could also significantly induce THP-1 cell apoptosis at 36 h post-treatment even without IFN-γ activation, although this phenomenon occurred to a lesser extent than that in IFN-γ-pretreated cells (Figures 1A,B).

We also applied lipopolysaccharide (LPS) for cell activation and found that LPS did not significantly influence the apoptosis-inducing effect of SEB (Figure 1C), although LPS can increase the expression of MHCII on dendritic cells (Casals et al., 2007).

**Figure 2:**
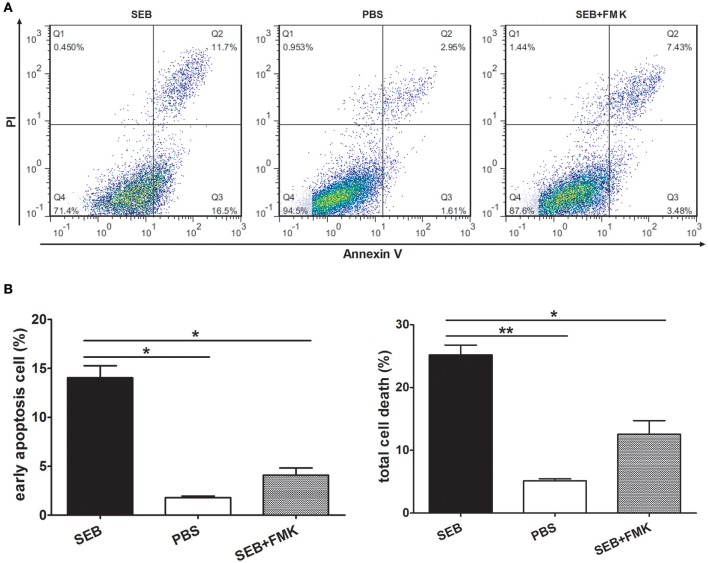
**SEB induced apoptosis in differentiated THP-1 cells**. Cells were inoculated in 24-well-plates (2 × 10^5^ per well) and pre-treated with 30 nM PMA for 48 h. After the medium was changed with a fresh culture medium, the cells were then treated with 20 μg/ml (24 μl/well) SEB with (striped bar) or without (black bar) 20 μM (2 μl/well) Z-VAD-FMK and 24 μl/well PBS (white bar) for 36 h. **(A)** Apoptosis was measured through Annexin V/PI staining and flow cytometry. **(B)** Quantitatively analyzed data are presented as means ± *S.D*. (*n* = 3), ^*^indicated *P* < 0.05 and ^**^indicated *P* < 0.01.

The proportion of apoptotic cells was even higher than that of the cells activated by IFN-γ when THP-1 cells were differentiated by 30 nM PMA before they were treated with SEB (Figures 2A,B). However, after the cells were activated by IFN-γ or differentiated by PMA, the control group (added with equal volume of PBS instead of SEB) obtained a higher apoptotic cell percentage than the non-activated or non-differentiated group, although without a statistical significance (Figures 1A,B, 2A,B). IFN-γ and PMA has been demonstrated to induce apoptosis in various cell types(Rosner et al., 2006; Javanmard and Dana, 2012; Kumbrink and Kirsch, 2013; Itsumi et al., 2014; Jamal et al., 2016), and IFN-γ could induce apoptosis in PMA-differentiated THP-1 cells (Inagaki et al., 2002). Therefore, we preferred to treat THP-1 cells directly with SEB in our further experiments to exclude the confusion on the actual effect of SEB with additional cytokines.

**Figure 3:**
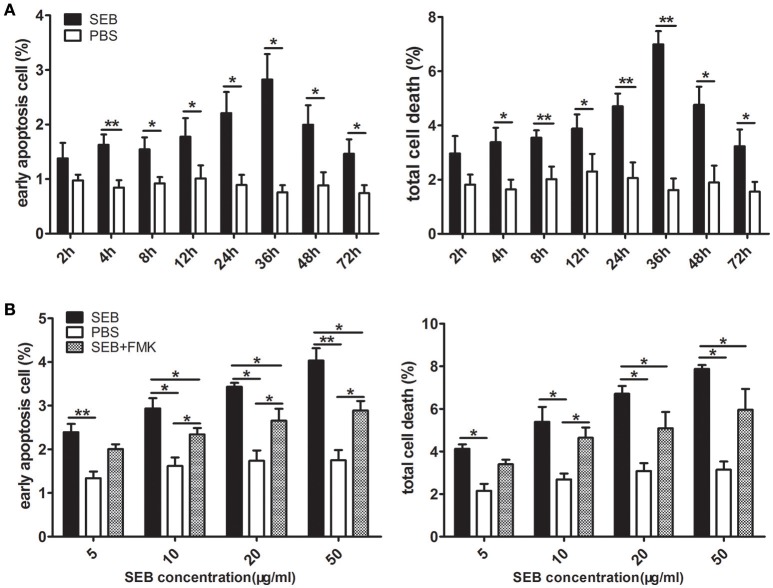
**SEB induced THP-1 cell apoptosis in a time- and dose-dependent manner. (A)** THP-1 cells (2 × 10^5^ per well in 24-well-plates) were treated with 20 μg/ml (24 μl/well) SEB (black bar) or 24 μl/well PBS (white bar) for 2, 4, 8, 12, 24, 36, 48, and 72 h. **(B)** THP-1 cells (2 × 10^5^ per well in 24-well-plates) were pre-treated with 20 μM (6 μl/well) Z-VAD-FMK for 30 min, and the cultures in the wells with 6 μl of RPMI 1640 medium containing 10% (ν/ν) DMSO served as controls. Cells were then treated with 5, 10, 20, or 50 μg/ml (60 μl/well) SEB (black bar and striped bar) or 60 μl/well PBS (white bar) for 36 h. Apoptosis was measured through Annexin V/PI staining and flow cytometry. Quantitatively analyzed data are presented as means ± *S.D*. (*n* = 4), ^*^indicated *P* < 0.05, and ^**^indicated *P* < 0.01.

When treated alone, SEB induced THP-1 cell apoptosis in a time- and dose-dependent manner (Figures 3A,B; Figures S1, S2). The percentage of apoptotic cells peaked at 36 h after treatment (Figure 3A; Figure S1). Therefore, we chose 36 h treatment for our further investigation. The proportion of apoptotic cells gradually increased as the SEB concentration was increased from 5 to 50 μg/ml (Figure 3B; Figure S2), and 20 μg/ml SEB was used in further experiments unless specifically stated. At this concentration, the irrelevant *S. aureus* proteins EsxA and EsxB were tested for their apoptosis-inducing effect in THP-1 cells, and the results showed no significant difference compared with control (data not shown).

### SEB induced apoptosis through an extrinsic caspase-dependent pathway

MHCII molecules can transduce signals to activate the PKC pathway and induce cell death in a caspase-independent manner (Thibeault et al., 1999; Castaigne et al., 2002). Considering this phenomenon, we determined whether SEB induces THP-1 cell apoptosis in the same manner. To test this, THP-1 cells were pretreated with Z-VAD-FMK, a caspase-specific inhibitor peptide, for 30 min before SEB treatment. The results showed that the apoptotic level of THP-1 cells was decreased at each concentration of SEB (Figure 3B; Figure S2). Furthermore, the treatment with Z-VAD-FMK also reduced the apoptotic level in PMA-differentiated THP 1 cells (Figures 2A,B). These findings indicated that SEB may induce cell apoptosis in a classical caspase-dependent pathway.

**Figure 4:**
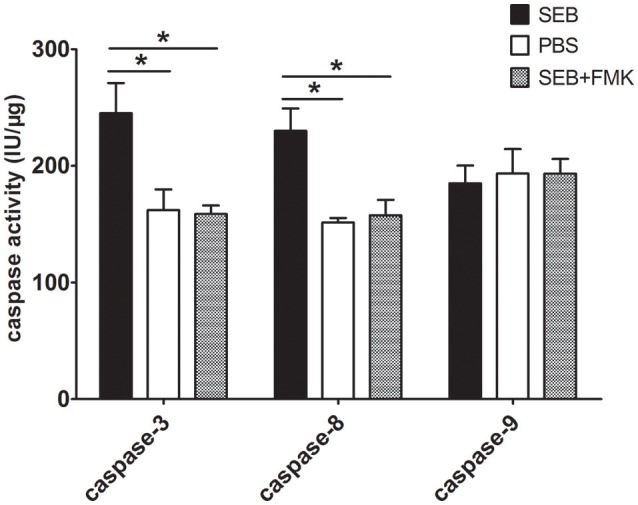
**SEB up-regulated caspase-3 and -8 activities, but not caspase-9 activity**. THP-1 cells (2 × 10^6^ per well in 6-well-plates) were pre-treated with 20 μM (20 μl/well) Z-VAD-FMK for 30 min, and the cultures in the wells with 20 μl of RPMI 1640 medium containing 10% (ν/ν) DMSO served as controls. Cells were then treated with 20 μg/ml (80 μl/well) SEB or 80 μl/well PBS for 36 h. After treatment, the cells were washed once with PBS and re-suspended in 100 μl of lysis buffer in an ice bath for 30 min. After centrifugation, the supernatants were transferred to 96-well-plates and incubated at 37°C for 2 h with each of the substrates for caspase-3, -8, and -9. The enzyme-catalyzed release of pNA was determined by using a spectrophotometer at 405 nm. For each sample, the total protein concentration was determined via Bradford's method. Caspase activity was calculated as international units per μg of sample protein concentration (IU/μg). Data are presented as means ± *S.D*. (*n* = 3), ^*^represented *P* < 0.05 vs. PBS control.

We determined the activities of caspase-3, -8, and -9 to confirm this observation and to explore SEB-induced apoptosis via either an extrinsic or an intrinsic pathway. After the cells were treated with SEB for 36 h, caspase-8 and -3 activities were obviously enhanced, whereas caspase-9 activity was not significantly changed. The activating effect of SEB on caspase-3 and -8 could also be impeded by Z-VAD-FMK (Figure 4). These results suggested that SEB induces THP-1 cell apoptosis via the extrinsic apoptosis pathway.

### SEB up-regulated the expression levels of TNFα and HLA-DRa

**Figure 5:**
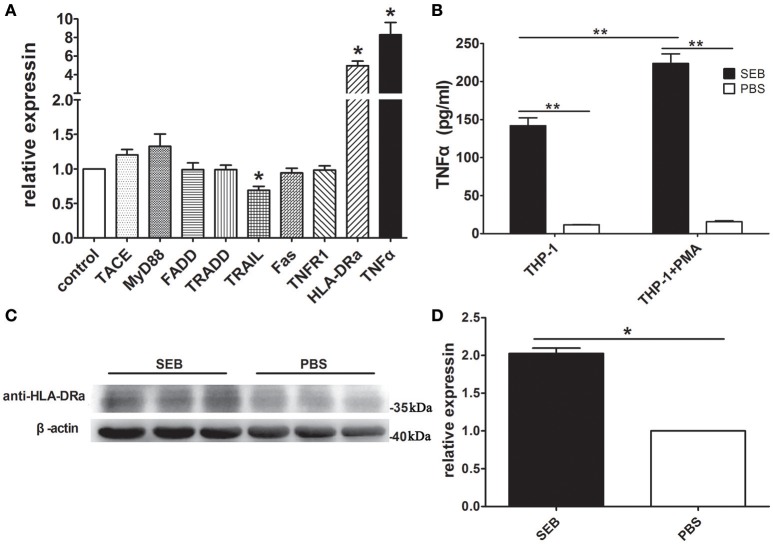
**SEB up-regulated TNFα and HLA-DRa expression. (A)** THP-1 cells (2 × 10^6^ per well) were inoculated in 6-well-plates and treated with 20 μg/ml SEB (80 μl/well) or 80 μl/well PBS for 36 h. qRT-PCR was conducted, as described in Materials and Methods. The mRNA levels of TACE, MyD88, TNFR1, HLA-DRa, TNFα, FADD, TRADD, TRAIL, and Fas were determined with their respective specific primers and normalized to the GAPDH expression. Data are presented as means ± S.D. (*n* = 4), ^*^represented *P* < 0.05 vs. PBS control. **(B)** THP-1 cells and PMA-differentiated THP-1 cells (2 × 10^5^ per well in 24-well-plates) were treated with 20 μg/ml (24 μl/well) SEB or 24 μl/well PBS for 36 h. The secreted TNFα in the culture supernatants was quantified through ELISA. Data are presented as means ± *S.D*. (*n* = 3), ^**^indicated *P* < 0.01 vs. PBS control. **(C)** THP-1 cells (2 × 10^6^ per well) were inoculated in 6-well-plates and treated with 20 μg/ml SEB (80 μl/well) or 80 μl/well PBS for 36 h. The HLA-DRa expression was determined through Western blot. **(D)** Quantitative analysis results with ImageJ software are presented as means ± *S.D*. (*n* = 3), ^*^represented *P* < 0.05 vs. PBS control.

SEB can induce TNFα expression (Kissner et al., 2011). As such, we hypothesized that SEB induces THP-1 cell apoptosis via TNFα. Quantitative real-time PCR (qRT-PCR) determination revealed that the mRNA level of TNFα was up-regulated after the THP-1 cells were treated with SEB. The mRNA levels of MyD88 and tumor necrosis factor-alpha convertase (TACE) were also up-regulated, although without a statistical significance (Figure 5A). Since MyD88 and TACE are crucial for TNFα expression (Caldwell et al., 2014), their enhancement may be responsible for the up-regulation of TNFα. It was interesting that the mRNA level of HLA-DRa was also increased significantly (Figure 5A), even known that TNFα can up-regulate MHCII expression (Krakauer and Oppenheim, 1993). This finding may explain why SEB could induce THP-1 cell apoptosis without cell activation. The up-regulated TNFα expression may in turn increase the HLA-DRa expression, which resembles the cell-activating effect of IFN-γ. However, the mRNA level of TNFR1, a TNFα receptor, was not changed significantly after SEB treatment was administered. The mRNA levels of other related proteins, such as FADD, TRADD, and Fas, were also not significantly altered, whereas the mRNA level of TRAIL was slightly but significantly down-regulated (Figure 5A). Thus, these proteins may not be involved in SEB-induced THP-1 cell apoptosis.

To further confirm these findings, we quantified the level of TNFα in the culture supernatant by ELISA and found that its concentration was consistent with the qRT-PCR results. The levels of TNFα in the supernatant of the SEB-treated THP-1 cells were significantly higher than that in the supernatant of the PBS-treated cells. This phenomenon also occurred in the PMA-differentiated THP-1 cells. When treated with SEB, the PMA-differentiated THP-1 cells showed even more secreted TNFα than naive cells (Figure 5B). These findings supported that SEB could up-regulate TNFα expression in THP-1 cells.

Western blot results confirmed that the HLA-DRa expression was up-regulated in the SEB-treated THP-1 cells. The HLA-DRa expression increased by approximately twofold when the cells were treated with SEB. This finding was consistent with the qRT-PCR results (Figures 5C,D).

### SEB-induced THP-1 cell apoptosisis dependent on a positive feedback cycle of TNF α

**Figure 6:**
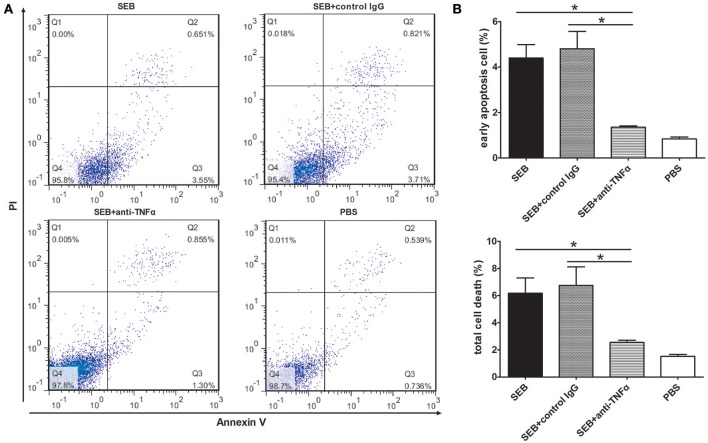
**Anti-TNFα neutralization reduced the SEB-induced apoptosis. (A)** THP-1 cells (2 × 10^5^ per well in 24-well-plates) were treated with 20 μg/ml (24 μl/well) SEB or 24 μl/well PBS for 36 h. For the neutralization group, 5 μg/ml anti-TNFα or an isotype control IgG was added 30 min before the SEB treatment was administered. Apoptosis was measured through Annexin V/PI staining and flow cytometry. **(B)** Quantitatively analyzed data are presented as means ± *S.D*. (*n* = 3), ^*^represented *P* < 0.05.

To further investigate the role of TNFα in SEB-induced apoptosis, we used an anti-TNFα antibody to neutralize the effect of TNFα. The apoptotic level of the THP-1 cells treated with a combination of SEB and anti-TNFα was remarkably lower than that of the cells treated with SEB alone or a combination of SEB and an isotype control IgG (Figures 6A,B).

**Figure 7:**
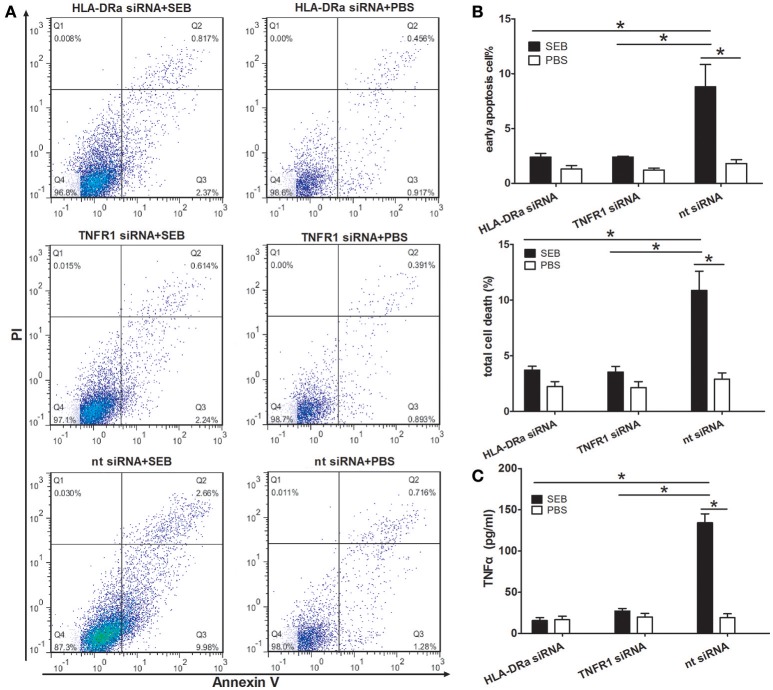
**SEB-induced apoptosis and TNFα expression were reduced in HLA-DRa- and TNFR1-knocked down THP-1 cells**. The siRNAs inhibition was conducted as described in Materials and Methods. HLA-DRa- and TNFR1-knocked down cells and control cells (2 × 10^5^ per well in 24-well-plates) were treated with 20 μg/ml (24 μl/well) SEB or 24 μl/well PBS for 36 h. **(A)** Apoptosis was measured through Annexin V/PI staining and flow cytometry. **(B)** Quantitatively analyzed data were presented as means ± *S.D*. (*n* = 4), ^*^indicated *P* < 0.05. **(C)** The secreted TNFα in the culture supernatants was quantified through ELISA. Data are presented as means ± *S.D*. (*n* = 3), ^*^indicated *P* < 0.05.

We next knocked down the HLA-DRa or TNFR1 expression in THP-1 cells by using siRNAs specific for the targets. The efficiency of siRNA inhibition was confirmed by Western blot. The expression levels of TNFR1 and HLA-DRa in the knocked down THP-1 cells were ~56 and 32% to that in the non-target siRNA control, respectively (Figures S3A,B). In the knocked down cells, the proportion of apoptotic cells induced by SEB was significantly decreased (Figures 7A,B), and this finding suggested that SEB-induced THP-1 cell apoptosis was dependent on TNFα expression and function. In addition, the TNFα expression levels were also decreased in the knocked down cells (Figure 7C), which may be a good explanation for the apoptosis reduction. Reduced TNFR1 may negatively influence the HLA-DRa expression because TNFα plays an important role in its regulation (Arenzana-Seisdedos et al., 1988). Therefore, the HLA-DRa expression could not be activated by TNFα in the TNFR1-knocked down cells. As a consequence, the effect of SEB was blocked. This observation indicated that TNFα functions as a receptor-activator and/or functional executor in SEB-induced THP-1 cell apoptosis.

## Discussion

MHCII molecules can transduce various extracellular signals, including apoptosis signals, which influence the process of diseases profoundly (Ong and Leung, 2010; Torchinsky et al., 2010; Aziz et al., 2014). Our study demonstrated that SEB, as a natural ligand of MHCII, could induce THP-1 cell apoptosis in a caspase-dependent manner. Considering that SEB could induce THP-1 cell apoptosis and up-regulate the caspase-8 and caspase-3 activities, but not caspase-9 activity, we concluded that the classical extrinsic pathway was involved in SEB-induced THP-1 cell apoptosis. This observation was further confirmed by the inhibition of caspase activity via Z-VAD-FMK, which remarkably reduced the SEB-induced apoptosis.Caspase-8, the initial proteinase of extrinsic apoptosis pathway, may not be directly activated by HLA-DRa because of the absence of known signaling motifs from the short cytoplasmic tail of MHC II molecules (Al-Daccak et al., 2004; Turner, 2004). Our results demonstrated that caspase-8 could be indirectly activated by TNFα, which was substantially secreted after SEB treatment. Both qRT-PCR and ELISA results confirmed that the TNFα expression increased remarkably when THP-1 cells were treated with SEB. However, the mRNA level of Fas was not significantly changed, and the mRNA level of TRAIL was slightly but significantly decreased. These findings suggested that these proteins may not be involved in SEB-induced THP-1 cell apoptosis. Nevertheless, further experiments should be conducted to confirm this conclusion.

The anti-TNFα antibody-induced neutralization of the secreted TNFα caused a significant decrease in the level of SEB-induced apoptosis. Therefore, TNFα was crucial for SEB-induced THP-1 cell apoptosis. The up-regulated TNFα expression can subsequently increase the HLA-DRa expression (Arenzana-Seisdedos et al., 1988). As a result, the effect of SEB could be amplified. On one hand, the ligation of SEB with MHCII up-regulated the TNFα expression; on the other hand, the up-regulated TNFα could in turn increase the HLA-DRa expression and thus create a positive feedback cycle which would likely promote THP-1 cell apoptosis. In this feedback cycle, SEB and TNFα are required to bind to their receptors because neither HLA-DRa- nor TNFR1-knocked down cells expressed a similar amount of TNFα or achieved the same level of apoptosis compared with that in the control cells. In contrast to mAb L243, SEB could induce THP-1 cell apoptosis even without cell activation possibly because of the increased TNFα expression induced by SEB and the formation of the positive feedback cycle of TNFα.

Our findings demonstrated that SEB induced THP-1 monocyte apoptosis via the TNFα-mediated extrinsic apoptosis pathway, and our findings differed from those described in previous studies, which demonstrated that human monocyte death is induced by mAb L243 against MHCII and is dependent on the activation of the PKC signal pathway (Thibeault et al., 1999; Castaigne et al., 2002). SEB induces T cell and epithelial cell apoptosis via Fas-mediated processes (Ulett and Adderson, 2006); however, this process is different from those observed in monocytes. Furthermore, even for a given cell type, apoptotic mechanisms may vary because of different stimulating toxin or molecular conditions (Ulett and Adderson, 2006). Therefore, apoptotic signal transduction is a complex process that should be further investigated.

Since SEB expression would change in different conditions, such as microbial content, salt, pH, nutrient availability, oxygen availability, and temperature (Schelin et al., 2011), it is hardly to determine a suitable concentration in cell models. Most studies on SEB applied the concentration from 0.1 to 50 μg/ml (Kędzierska et al., 2005; Ionin et al., 2008; Kissner et al., 2011), and we tested concentrations ranged from 5 to 50 μg/ml and all the concentrations were efficient to induce THP-1 cell apoptosis. Although the positive feedback cycle of TNFα is crucial for SEB functions, the mechanism by which SEB up-regulates TNFα expression is poorly understood. Previous studies elucidated that MyD88 and TACE are responsible for SEB-induced TNFα expression (Khan et al., 2008; Kissner et al., 2011). Their mRNA levels were up-regulated in our experiments, but the upper regulatory molecules have yet to be determined. Since MHCII contains a relatively short cytoplasmic tail without a predicted motif and membrane receptors couple with MHCII for signal transduction (Castaigne et al., 2002; Lévéille et al., 2002), it is reasonable to assume an additional protein which coupled with HLA-DRa to trigger apoptosis signals induced by SEB. This phenomenon should also be further investigated. Although MHCII expression is exclusively controlled by a class II transactivator (CIITA) and IFN-γ regulates CIITA transcription via a STAT1 pathway (Reith et al., 2005), the mechanism by which TNFα interacts with CIITA remains unknown (Arenzana-Seisdedos et al., 1988; Ishii et al., 1994). Moreover, whether TNFα is the only signaling molecule that triggers SEB-induced THP-1 cell apoptosis has yet to be verified because HLA-DRa activation stimulated by TNFα is essential for SEB functions. Nevertheless, we may conclude that TNFα is the activator of HLA-DRa and is crucial for the positive feedback cycle that promotes SEB-induced THP-1 cell apoptosis.

In clinical settings, AD severity is correlated with SEB-induced apoptosis (Kędzierska et al., 2005), and therapy with infliximab, a TNFα inhibitor, significantly improves clinical parameters, although this improvement is not sustained (Jacobi et al., 2005). Thus, TNFα may be implicated in early stages of AD pathogenesis. For the therapy of staphylococcal superantigen-induced shock, neutralizing antibodies against TNFα prevents SEB-induced lethality in a mouse model and thus confirms the critical role of TNFα in SEB-induced shock (Krakauer, 2010).

In conclusion, our study demonstrated that TNFα functions as an activator for the SEB receptor might also an executer in SEB-induced THP-1 cell apoptosis. This process is dependent on the formation of a positive feedback cycle of TNFα, which is crucial for the pathogenic function of SEB. These findings emphasized the importance of TNFα in diseases induced by SEB, but further studies on drug development should be performed.

## Author contributions

XZ, XH, and XR conceived and designed the experiments. XZ, WS, JY, HP, HL, BJ, and YW performed the experiments. XZ, SL, QH, YY, and XR analyzed the data. JZ and ZH contributed reagents/materials/analysis tools. XZ, XH, and XR wrote the paper.

## Funding

This work was supported by National Natural Science Foundation of China (grants no. 31470241 and 31570127).

### Conflict of interest statement

The authors declare that the research was conducted in the absence of any commercial or financial relationships that could be construed as a potential conflict of interest.
