# Supplementary material for: Positive Feedback Cycle of TNFα Promotes Staphylococcal Enterotoxin B-Induced THP-1 Cell Apoptosis
Source: Front Cell Infect Microbiol. 2016 Sep 21;6:109. doi: 10.3389/fcimb.2016.00109 (PMC5030291; doi:10.3389/fcimb.2016.00109)
Supplement: Supplementary file 1 [file Table1.DOCX]

Supplementary Materials

### Positive feedback cycle of TNFα promotes staphylococcal enterotoxin B-induced THP-1 cell apoptosis

**Xiaopeng Zhang, Weilong Shang, Jizhen Yuan, Zhen Hu, Huagang Peng, Junmin Zhu, Qiwen Hu, Yi Yang, Hui Liu, Bei Jiang, Yinan Wang, Shu Li, Xiaomei Hu*, Xiancai Rao***

*** Correspondence:** Xiaomei Hu: hxmay2008@163.com;

Xiancai Rao: raoxiancai@126.com

# Table S1 The primers used in qRT-PCR

| target gene | Sequence | size | reference |
| --- | --- | --- | --- |
| TNFα | F: 5′-AGAAGGGTGACCGACTC-3′ | 123bp | this study |
|  | R: 5′-GTTTGGGAAGGTTGGAT-3′ |  |  |
| TACE | F: 5′-GGCAAATGTGAGAAACGAG-3′ | 120bp | this study |
|  | R: 5′-AACCAGGACAGACCCAACG-3′ |  |  |
| MyD88 | F: 5′-CTCTGGAAAGGACCCAATGTA-3′ | 257bp | this study |
|  | R: 5′-GCTGAAAGTGGAGCAAAGATA-3′ |  |  |
| TNFR1 | F: 5′-CTGCCAGGAGAAACAGAACA-3′ | 244bp | this study |
|  | R: 5′-CCGTTGGTAGCGATACATTA-3′ |  |  |
| HLA-DRa | F: 5′-TCTTGTCTGTTCTGCCTCACT-3′ | 209bp | this study |
|  | R: 5′-AAACATAAACTCGCCTGATTG-3′ |  |  |
| FADD | F: 5′-GTGAAGACCCAGCAGGAAGC-3′ | 369bp | this study |
|  | R: 5′-ACGCCACAGTGGTTGAGCAT-3′ |  |  |
| TRADD | F: 5′-TCTGCGGCTATTGCTGAAC-3′ | 211bp | this study |
|  | R: 5′-CTGGGATGGGAGAAGGTGA-3′ |  |  |
| TRAIL | F: 5′-TTGGCTAACTGACCTGGAA-3′ | 166bp | this study |
|  | R: 5′-TTTGGTTGTGGCTGCTCTA-3′ |  |  |
| Fas | F: 5′-TTCTGCCATAAGCCCTGTC-3′ | 322bp | this study |
|  | R: 5′-ACTTGGTGTTGCTGGTGAG-3′ |  |  |
| GAPDH | F: 5′-AACGGATTTGGTCGTATTGGGC-3′ | 216bp | ([Wang et al., 2013](#_ENREF_1)) |
|  | R: 5′-TCGCTCCTGGAAGATGGTGAT-3′ |  |  |

# Reference:

Wang Q., Zhou J., Zhang B., Tian Z., Tang J., Zheng Y., Huang Z., Tian Y., Jia Z., Tang Y., Van Velkinburgh J.C., Mao Q., Bian X., Ping Y., Ni B., and Wu Y. (2013). Hepatitis B virus induces IL-23 production in antigen presenting cells and causes liver damage via the IL-23/IL-17 axis. *PLoS Pathog* 9 (6):e1003410.doi:10.1371/journal.ppat.1003410

# Figure legends

# Figure S1

SEB induced THP-1 cell apoptosis in a time-dependent manner. THP-1 cells (2×10^5^ per well in 24-well plates) were treated with 20 µg/ml (24 µl/well) SEB or 24 µl/well PBS for 2, 4, 8, 12, 24, 36, 48, and 72 h. Apoptosis were measured by Annexin V/PI staining and flow cytometry. Cells stained with Annexin V+/PI− were considered as early apoptotic cells, and Annexin V+/PI+ were regarded as late apoptosis and necrotic cells. Total cell death corresponded to all of the cells positively stained with PI.

# Figure S2

SEB induced THP-1 cell apoptosis in a time- and dose-dependent manner. (B) THP-1 cells (2×10^5^ per well in 24-well plates) were pre-treated with 20 µM (6 µl/well) Z-VAD-FMK for 30 min, and the cultures in the wells with 6 µl of RPMI 1640 medium containing 10% (*v*/*v*) DMSO served as controls. Cells were then treated with 5, 10, 20, or 50 µg/ml (60 µl/well) SEB or 60 µl/well PBS for 36 h. Apoptosis was measured through Annexin V/PI staining and flow cytometry. Cells stained with Annexin V+/PI− were considered as early apoptotic cells, and Annexin V+/PI+ were regarded as late apoptosis and necrotic cells. Total cell death corresponded to all of the cells positively stained with PI.

# Figure S3

The confirmation of siRNA inhibition efficiency. A total of 32 µl of siRNAs (HLA-DRa siRNA, TNFR1 siRNA, or non-target control siRNA; 10 µM) were diluted in 400 µl of transfection medium, mixed with 400 µl of transfection medium containing 24 µl of transfection reagent (856 µl for each siRNA reagent), and incubated in the dark at room temperature for 30 min. The cells (2×10^6^) were washed once with 2 ml of transfection medium, re-suspended in the siRNA reagent, and incubated at 37 °C for 6 h in a CO_2_ incubator. The siRNA reagent was removed and replaced with 2 ml of fresh culture medium for another 24 h. (A) The inhibition efficiency was confirmed by Western blot. The cells were collected and analyzed by Western blot with anti-HLA-DRa (upper panel) and anti-TNFR1 (bottom panel). The β-actin served as loading control, and the protein sizes are indicated on the right. (B) Quantitatively analyzed data are presented as means ± S.D. (*n*=3), * indicated *P*< 0.05 and ** indicated *P*< 0.01 versus the non-target siRNA control.
