# Supplementary figures and images for: Positive Feedback Cycle of TNFα Promotes Staphylococcal Enterotoxin B-Induced THP-1 Cell Apoptosis
Source: Front Cell Infect Microbiol. 2016 Sep 21;6:109. doi: 10.3389/fcimb.2016.00109 (PMC5030291; doi:10.3389/fcimb.2016.00109)

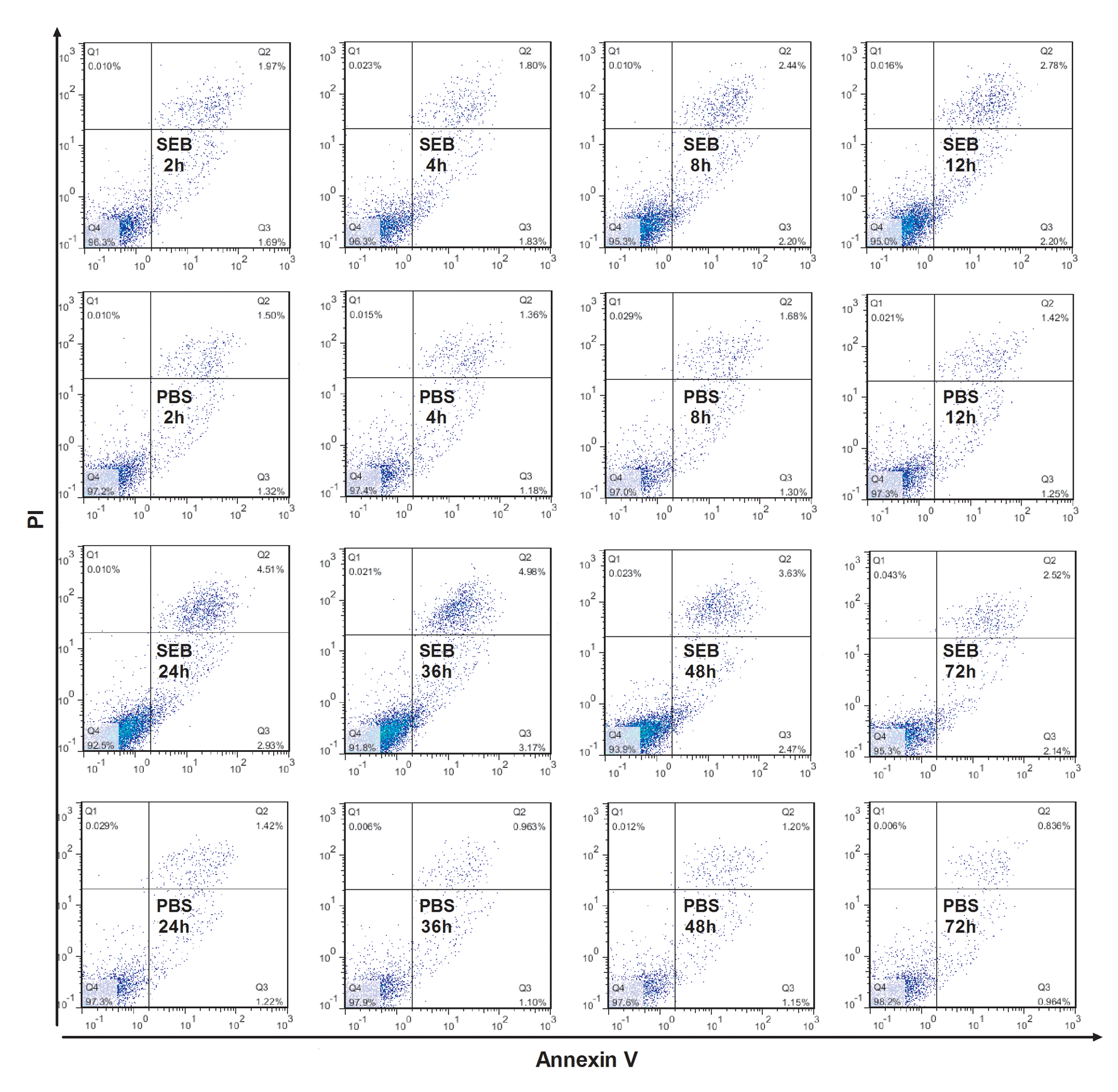

Supplement: Supplementary file 2 [file Image1.TIF]

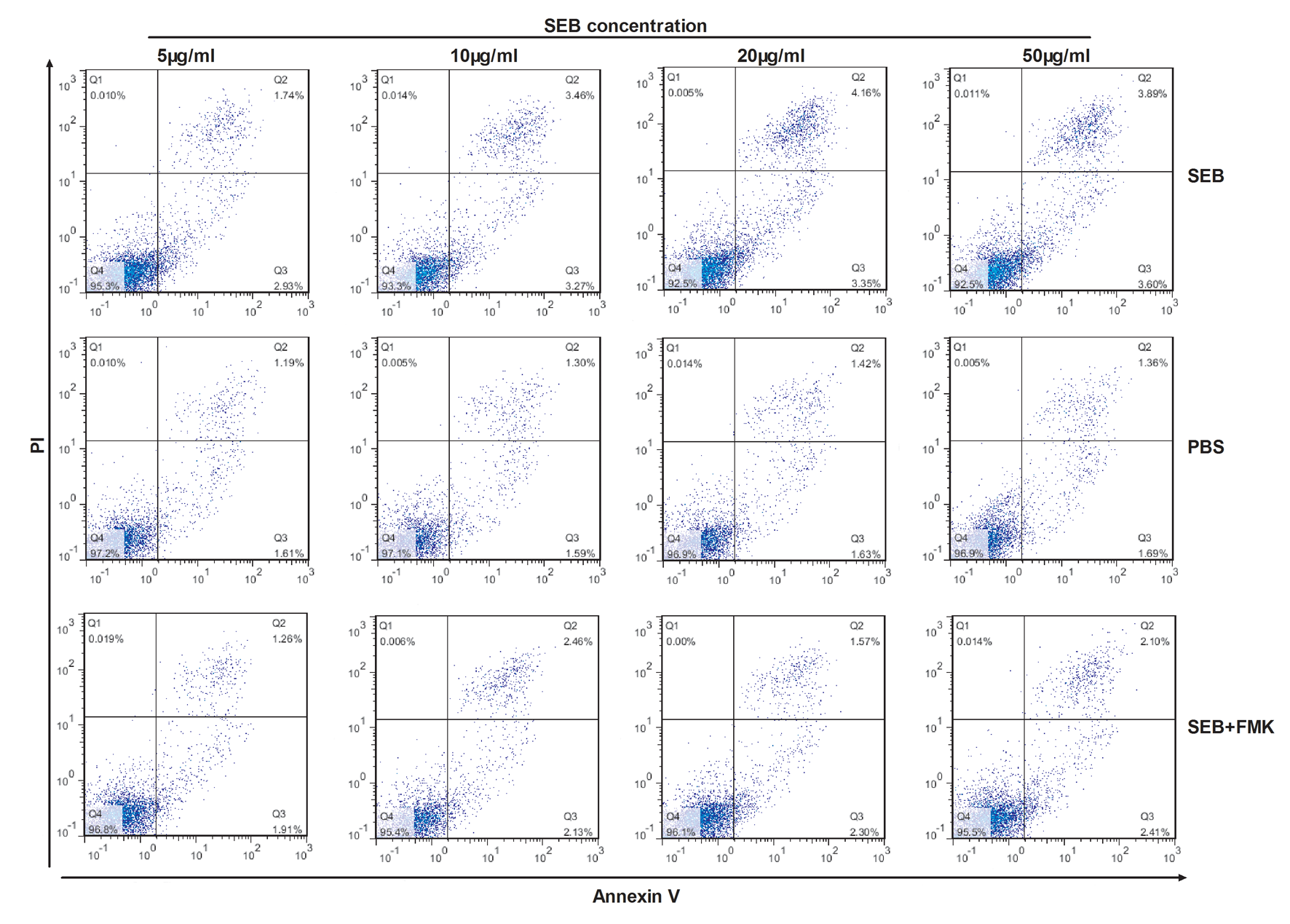

Supplement: Supplementary file 3 [file Image2.TIF]

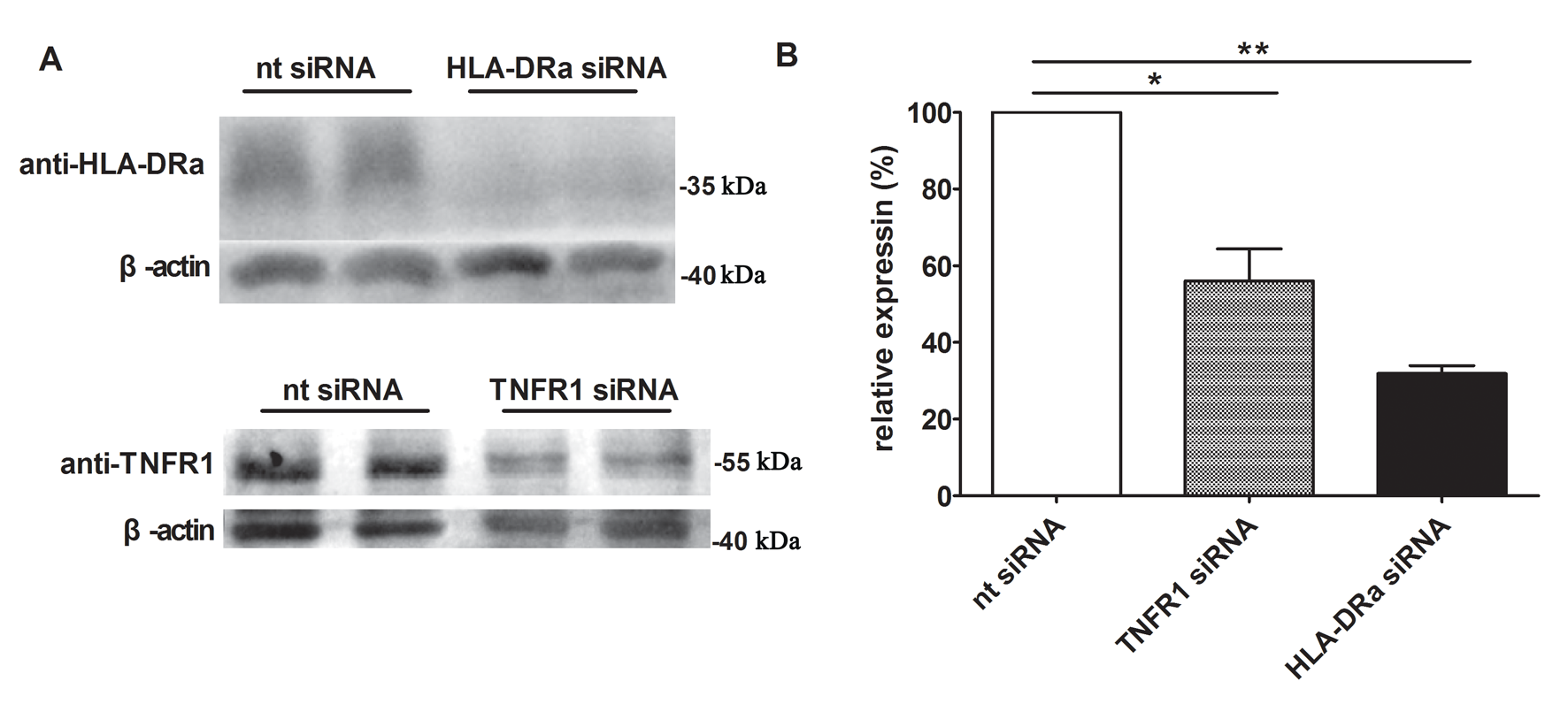

Supplement: Supplementary file 4 [file Image3.TIF]
